# Robust GICP-Based 3D LiDAR SLAM for Underground Mining Environment

**DOI:** 10.3390/s19132915

**Published:** 2019-07-01

**Authors:** Zhuli Ren, Liguan Wang, Lin Bi

**Affiliations:** 1 School of Resources and Safety Engineering, Central South University, Changsha 410083, China; 2 Digital Mine Research Center, Central South University, Changsha 410083, China

**Keywords:** underground mine, SLAM, GICP, graph optimization, roadway plane, loop detection

## Abstract

Unmanned mining is one of the most effective methods to solve mine safety and low efficiency. However, it is the key to accurate localization and mapping for underground mining environment. A novel graph simultaneous localization and mapping (SLAM) optimization method is proposed, which is based on Generalized Iterative Closest Point (GICP) three-dimensional (3D) point cloud registration between consecutive frames, between consecutive key frames and between loop frames, and is constrained by roadway plane and loop. GICP-based 3D point cloud registration between consecutive frames and consecutive key frames is first combined to optimize laser odometer constraints without other sensors such as inertial measurement unit (IMU). According to the characteristics of the roadway, the innovative extraction of the roadway plane as the node constraint of pose graph SLAM, in addition to automatic removing the noise point cloud to further improve the consistency of the underground roadway map. A lightweight and efficient loop detection and optimization based on rules and GICP is designed. Finally, the proposed method was evaluated in four scenes (such as the underground mine laboratory), and compared with the existing 3D laser SLAM method (such as Lidar Odometry and Mapping (LOAM)). The results show that the algorithm could realize low drift localization and point cloud map construction. This method provides technical support for localization and navigation of underground mining environment.

## 1. Introduction

With the rapid development of ground unmanned driving and the harsh environment of deep resource exploitation, in order to improve the safety status of underground transportation operations and maximize the economic benefits of mining enterprises [1], the unmanned of underground mining environment is an inevitable trend in the future development. The intelligent and precise localization of underground mining environment is the key. However, it is impossible to use GPS signals to locate them in the special environment of underground confined spaces such as underground mines and subways. As early as the early 1990s, low frequency electromagnetic, ultrasonic sensing measurements, and visual beacon navigation were used for underground positioning and continuous tracking [2,3,4]. Localization technologies based on WiFi, Bluetooth, Radio Frequency IDentification (RFID), Ultra Wideband (UWB), and ultrasound are also widely used [5]. However, in the above localization and mapping methods, the corresponding auxiliary localization devices need to be installed in the underground environment. Although the positioning accuracy can be improved, a large amount of devices and maintenance costs are required. At the same time, due to the rough underground environment, it is easy to cause a cumulative error during the localization process. Especially, it often fails to get the pose in the case of rotation, which greatly reduces the safety during the autonomous walking of the trackless mining equipment.

With the development of computer performance and related optimization algorithms, simultaneous localization and mapping (SLAM) technology [6] is becoming more and more mature. It provides an important reference for the intelligent localization and mapping of underground metal mines, and then realizes path planning and autonomous navigation of trackless mining equipment. Although SLAM technology has made remarkable progress in the past 30 years, for a closed underground special environment, the roadway surface is uneven and degraded over time, which brings difficulties to the application of laser SLAM. At the same time, due to the dust and poor lighting conditions in the underground environment, it causes the feature point extraction to be unstable and fails in the visual SLAM. In general, the solution of laser SLAM has a larger application space in underground mines.

The two-dimensional (2D) laser-based SLAM has lower computational requirements and the map can be built in real time in the scan plane. However, it cannot estimate the six-degree-of-freedom pose of a mobile robot in three-dimensional (3D) space on uneven ground. Another disadvantage is that long roadways with high similarity are difficult to match accurately because there are too few features available in one scan plane; Huber and Vandapel [7] used the stop-scan-start method to convert 3D laser information into a unified world coordinate system, and then constructed a high-precision 3D geological model of the mine roadway. However, high-precision laser scanners are expensive, and it cannot meet the real-time mapping and localization requirements of underground mining environment. Thus, many SLAM systems combine laser scanners with other sensors for greater precision and robustness. Lopez et al. [8] integrated 2D laser, vision, altimeter, and inertial measurement unit (IMU) to improve the accurate acquisition of six degrees of freedom (6DoF) pose estimation of robots in environments where GPS signals cannot be received. Bosse et al. [9] coupled a 2D laser to an inertial measurement unit mounted on a spring. This method is well adapted for strenuous motion, but it is suitable for mapping and is not suitable for robot positioning.

The current 3D SLAM method is usually computationally intensive, and it is difficult to achieve real-time operation under limited computing resources [7,10,11]. Since there is no real-time method to reduce cumulative error, the laser-based methods most focuses on the point cloud registration process or it is only used in laser odometers, which result in poor adaptability in large-scale and rough ground environments [12]. Aiming at the shortcomings of traditional algorithms, this paper mainly studies the state estimation and environmental mapping of underground mining environment by using 3D laser, which provides support for intelligent mining of underground mines.

There are four contributions in this paper: Firstly, Generalized Iterative Closest Point (GICP)-based 3D point cloud registration between consecutive frames and consecutive key frames is first combined to optimize laser odometer constraints, which plays a major role in the unstructured environment. Secondly, a fast point cloud segmentation based on RANdom Sample Consensus (RANSAC) is used to extracts the roadway plane, which serves as a landmark to construct the observation constraint in the graph SLAM optimization. Thirdly, a lightweight and efficient loop detection and optimization based on rules and GICP is designed, which is applied to correct motion drift in pose graph optimization. Fourthly, for the constructed sub-map, automated removal of point cloud noise based on the characteristics of the roadway is first proposed, and then the completed point cloud map can be widely applied, such as 3D point cloud modeling and positioning based on the known maps.

In addition, our results show that a complete 3D LiDAR graph optimized SLAM framework has wider applicability, and it provides technical support for the practical application of special environments in underground confined spaces such as subways, corridors, tunnel fire-fighting, and civil air defense works.

The rest of the paper is organized as follows: Section 2 describes the related work; Section 3 describes the proposed method in detail; Section 4 presents an experiment and analysis on underground mining environment; and in the last section, we present conclusions and recommendations for future work.

## 2. Related Work

The SLAM method based on laser has been the cornerstone of mobile robot mapping and navigation research for the past 20 years. At the same time, relying on the constructed 3D map for 6D localization is still a research hotspot. Compared with vision sensors, LiDAR provides measurement information that is more robust, accurate, and noise level stable, and is not sensitive to changes in illumination conditions. Therefore, laser SLAM is the most stable and reliable SLAM solution. Most of the work on 3D lasers is frames matching, which is used to predict the relative transformation between two frames. Laser SLAM can be divided into filter-based and graph-based optimization according to different solution methods. The laser SLAM system framework based on graph optimization is currently popular. It is mainly divided into two parts: front end and back end. The front end completes data association and loop detection, and the back end performs graph optimization.

Laser scan matching is the most common method for achieving laser SLAM data correlation. It is defined as a set of translation and rotation parameters, so that the aligned two-frame scanning point cloud reaches the maximum overlap. Laser scan matching is divided into three categories: (1) point-based scan matching; (2) feature-based scan matching; and (3) scan matching based on mathematical characteristics.

### 2.1. Point-Based Scan Matching

Point-based scan matching is performed directly on the raw data points acquired by the scan, and the Iterative Closest/Corresponding Point (ICP) algorithm is proposed by Chen [13] and Besl [14] alone, respectively. It is the most widely used, most researched and currently the most mature algorithm. The difference is that the former uses the point-to-surface distance as the error metric, while the latter uses the point-to-point distance as the error metric, so it can be recorded as Point to Plane ICP and Plane to Plane ICP. Combined with Point-to-Plane ICP algorithm and Plane to Plane ICP algorithm in a single probability framework, GICP algorithm [15] and its improved algorithm [16,17] is proposed. It has become one of the most effective and robust algorithms in many ICP improved algorithms, especially in indoor and structured scenarios where GICP performs better than standard ICP.

### 2.2. Feature-Based Scan Matching

Feature-based scan matching methods are usually based on flexible features such as normal and curvature, as well as custom feature descriptors. Such as the HSM (Hough Scan Matching) method [18], using the Hough transform to extract line segment features and matching in the Hough domain. By using the Loam (LiDAR odometry and mapping) algorithm [19] and its improved algorithm [20], the LiDAR odometer is realized by matching feature points to edge segments and planes, and excellent results have been achieved in various scenarios.

### 2.3. Scan Matching Based on Mathematical Characteristics

In addition to point-based scan matching and feature-based scan matching, there are a large class of scan matching methods that use various mathematical properties to characterize scan data and frames pose changes, the most famous of which is based on Normal Distributions Transform (NDT) [21] and its improved algorithm [12].

Inevitably, pose accumulative errors will occur only when pose estimation is considered in adjacent time, and it is impossible to obtain globally consistent trajectories and maps. Graph optimization is an effective method to reduce cumulative errors and is widely used in SLAM back-end optimization [22]. Graph-based SLAM usually uses robot poses and landmarks in the environment as state variables. The nodes in the graph are the variables to be optimized, and the edges are the observation constraints between the two interconnected variables. Bundle adjustment (BA) [23] is a classical optimization method based on visual SLAM. It takes the sensor pose and landmarks in the environment as optimization variables, and reduces the size based on sparse features. Key frame nodes formed by the sensor pose are considered in the pose optimization [24], thereby saving optimization time for many features.

Loop detection [25] finds correlation between current data and all historical data. More constraints are provided to build a globally consistent mapping in the graph optimization. Loop detection in degraded scenes such as underground roadways is still difficult and time consuming due to small differences in texture and features. The word ‘bag’ [26] is a common method in visual SLAM, which uses feature clustering to construct a dictionary and detect similarities between two image frames. In Google’s Cartographer [27], the branch and bound method is applied to frame and sub-map matching to create loop constraints. Magnusson [28] proposed an appearance-based loop method that created a feature histogram of the surface topography. Two frame scans are effectively matched and good results are obtained.

## 3. Simultaneous Localization and Mapping Framework

### 3.1. Overview

**Figure 1:**
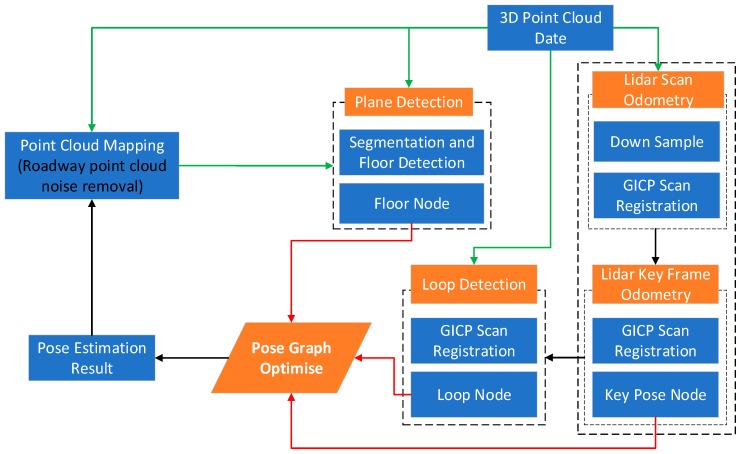
Generalized Iterative Closest/Corresponding Point (GICP)- simultaneous localization and mapping (SLAM) system framework.

Based on GICP with high registration accuracy, the 3D point cloud registration between consecutive frames, consecutive key frames and loop frames is used to calculate the transformation pose. Graph optimized SLAM for underground mining environment is constructed with key pose, roadway plane and loop as constraints. An overview of the proposed algorithm framework is given in Figure 1, which is based on four main parallel threads: LiDAR odometer, plane constraint, loop constraint and pose optimization. As shown by the orange background in the flowchart. The green arrow indicates the input of the 3D point cloud information, the black arrow indicates the input of the laser odometer information, and the red arrow indicates the node constraint information input in the pose graph optimization. Finally, the optimized pose information and the point cloud map information after noise removal are obtained. GICP-based frame and frame registration result is the initial value of the registration between key frames, and GICP is used again to optimize the key frame, and the optimized key frame pose is used as the pose node of graph SLAM. According to the characteristics of the roadway, the ground plane and loop detection frame are innovatively extracted as the pose constraint optimization constraints, and the cumulative error caused by the laser odometer is reduced.

### 3.2. GICP Method Description

GICP is based on the addition of a probability model to the standard ICP minimization step, with the rest remaining unchanged. The standard Euclidean distance is used instead of the probability measure to calculate the correspondence, thus maintaining the principle advantage of GICP over other fully probabilistic techniques.

For a two-frame point cloud $A=\{a_{i}\},B=\{b_{i}\}$, the transformation matrix between the two frame point clouds is T. The B point cloud is converted to the A point cloud according to the transformation matrix, and then the corresponding nearest point $M=\{m_{i}\}$ is found under the A point cloud. There are two frames of point cloud $\hat{M}=\{{\hat{m}}_{i}\},\hat{B}=\{{\hat{b}}_{i}\}$ in the probability model, and the points in the point cloud M, B obey the distribution $m_{i}∼Ν({\hat{m}}_{i},C_{i}^{M})$, $b_{i}∼Ν({\hat{b}}_{i},C_{i}^{B})$, and the correct transformation matrix $T^{*}$ between the two frames satisfies the Formula (1):

(1) $${\hat{m}}_{i}=T^{*}{\hat{b}}_{i},$$

where $C_{i}^{M}$ and $C_{i}^{B}$ are covariance matrices. For a point $p_{i}$ in a given point cloud P, Formula (2) is used to calculate its mean and covariance matrix:

(2) $${\bar{x}}_{i}=\frac{1}{k}\sum_{i=1}^{k}p_{i},C=\frac{1}{k}\sum_{i=1}^{k}(p_{i}-{\bar{x}}_{i})\cdot {\left(p_{i}-{\bar{x}}_{i}\right)}^{T}.$$

At the same time, the Singular Value Decomposition (SVD) decomposition of the covariance matrix C is performed to obtain the U,V matrix. To ensure that each observation point only provides constraints along its surface normal, the singular value matrix is set to Σ=diag(ε,1,1), where is a fixed constant much less than 1, usually 0.001. The final covariance matrix can be obtained by using the Formula (3):

(3) $$C_{i}^{M}=U_{i}^{M}\Sigma U_{i}^{MT},C_{i}^{B}=U_{i}^{B}\Sigma U_{i}^{BT}.$$

For a rigid body transformation matrix T, Formula (4) is the distance between two registration point clouds:

(4) $$d_{i}^{T}=m_{i}-Tb_{i}.$$

Then, Formula (5) is the Gaussian distribution of the point cloud distance:

(5) $$d_{i}^{T*}∼Ν({\hat{m}}_{i}-T*{\hat{b}}_{i},C_{i}^{M}+T*C_{i}^{B}T{*}^{T})=Ν(0,C_{i}^{M}+T*C_{i}^{B}T{*}^{T}).$$

Using Formula (6), the transformation matrix can be obtained from the maximum likelihood estimation:

(6) $$\begin{array}{l} T\propto \underset{T}{\arg\;\max}\prod_{i}p(d_{i}^{T})\propto \underset{T}{\arg\;\max}\sum_{i}\log(p(d_{i}^{T}))\\ \propto \underset{T}{\arg\;\max}\sum_{i}{\left(d_{i}^{T}\right)}^{T}{\left(C_{i}^{M}+T*C_{i}^{B}T{*}^{T}\right)}^{-1}(d_{i}^{T}) \end{array}$$

### 3.3. Laser Odometer

#### 3.3.1. Laser Odometer between Consecutive Frames

In the proposed system, the LiDAR pose is first estimated by applying GICP scan matching between consecutive frames. For 3D LiDAR, GICP exhibits more reliability than other scan matching algorithms (such as ICP). There is almost no new information and accumulated linear error when processing the matching of consecutive frames. The GICP can be used to match the real-time input point cloud with the previous time frame to obtain the relative pose $\Delta T_{t-1,t}$ from t to t−1, and the point position of the cloud at t−1 is $T_{t-1}$. Therefore, the 3D LiDAR pose at time t can be calculated by the following Formula (7):

(7) $$T_{t}=T_{t-1}\Delta T_{t-1,t}.$$

By continuously using GICP for iteration, the pose of the 3D laser at all times can be obtained.

#### 3.3.2. Laser Odometer between Consecutive Key Frames

**Figure 2:**
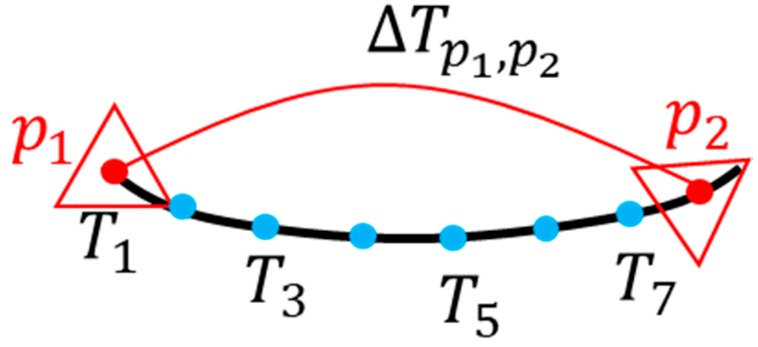
Schematic diagram of laser odometer between consecutive key frames.

The key frame concept was originally used for visual SLAM, which can greatly improve computational efficiency. Especially it can ensure that the algorithm can run in real time for larger environmental maps. When selecting key frames, it is necessary to reduce the matching error while reducing redundant key frames to save calculations. This is because the sparse key frames will lead to increased uncertainty of observation between frames, and it is very unfavorable in optimization. The map quality is poor. According to the key frame selection method of the visual SLAM and the first frame point, the cloud, is used as the key frame. The key frame selection is performed based on the key frame judgment criterion for the real-time point cloud. According to the odometer between the consecutive frames, the pose $T_{k-1}$ of the k−1 key frame and the pose of the k key frame are $T_{k}$. As shown in Figure 2, the relative transformation pose of the continuous key frame can be obtained by using the Formula (8):

(8) $$\Delta T_{k-1,k}=T_{k-1}^{-1}T_{k}=\left(\begin{array}{cc} \Delta R & \Delta s\\ 0 & 1 \end{array}\right).$$

The Euclidean distance between consecutive key frames is $\left\|\Delta s\right\|=\sqrt{\Delta x^{2}+\Delta y^{2}+\Delta z^{2}}$, the rotation between consecutive key frames is $\Delta r=\arccos(\frac{trace(\Delta R)-1}{2})$, and the criterion for the key frame is that when any of the three conditions ‖Δs‖, Δr, and time Δt exceeds a set threshold, then The point cloud is the key frame.

After determining the key frame, the GICP registration algorithm is used again to optimize the point cloud registration with the initial relative transformation pose. The final transformed pose is applied to the constraint between the pose and the pose in the pose graph. The pose node is the sensor pose obtained by the above laser continuous frame.

### 3.4. Graph SLAM Optimization

#### 3.4.1. Loop Detection

**Figure 3:**
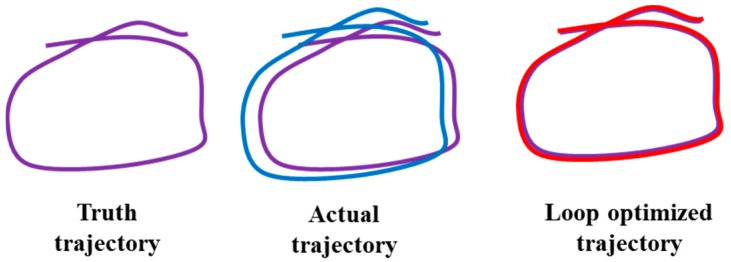
Schematic diagram of the loop optimization process.

Consecutive frames and consecutive key frames laser odometer only consider the correlation between adjacent time and adjacent key frames. However, the error generated by the previous state will inevitably accumulate to the next state, so that the cumulative error will occur in the whole SLAM. Long-term estimates will be unreliable and will not be able to construct globally consistent trajectories and maps, as shown in Figure 3.

**Figure 4:**
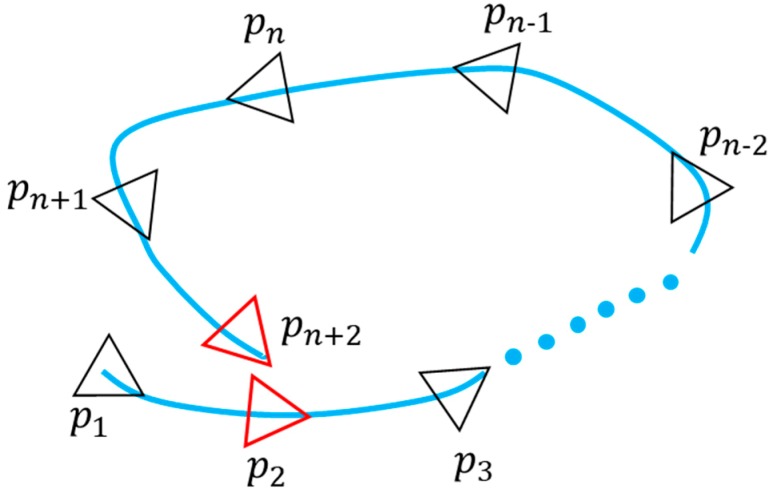
Schematic diagram of loop detection.

When performing loop detection, first compare the current real-time key frame with the historical key frame, and then select the candidate loop frame. The following conditions are met, as shown in Figure 4:

1. The index of the current key frame is larger than the index of the historical key frame;
2. The difference between the trajectory distances of the current key frame and the historical key frame is greater than a set threshold;
3. The relative translation distance between the current key frame and the historical key frame is less than a set threshold.

Finally, according to the obtained candidate loop frame and the current key frame for GICP registration, the highest GICP registration score is selected as the final loop frame. The key frame and the loop frame are added as nodes to the graph SLAM optimization, and the edge constraint is the relative pose obtained by registration.

#### 3.4.2. Roadways Plane Detection

**Figure 5:**
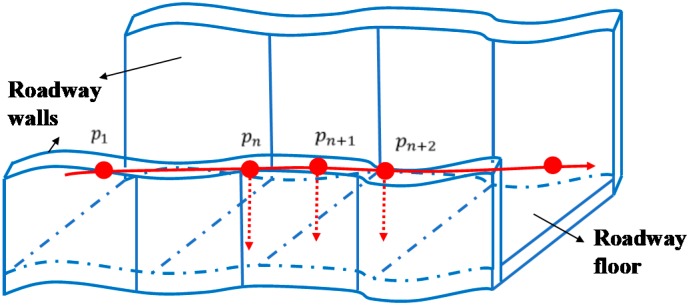
Schematic diagram of roadway plane constraint.

The underground mine environment is usually dominated by roadways of equal distance, as shown in Figure 5. When the robot is operating in different spatial regions, the tunnel plane can provide additional constraints for motion estimation of key frames. The same planar nodes can be observed under different pose nodes. The purpose of establishing planar constraints is to provide additional nodes to optimize the pose graph (similar to loop detection) while reducing the cumulative error by using more constraint information to improve the accuracy of pose estimation.

The key to establishing the plane constraint is to accurately extract the roadway plane, and choose a robust estimation method with less iterations and strong resistance to gross error (noise data)—Random Sample Consensus (RANSAC) [29]. The commonly used plane equation is the normal of the plane: ax+by+cz=d, where $a^{2}+b^{2}+c^{2}=1,d>0$, (a,b,c) is the plane normal vector, and d is the distance from the origin to the plane. These four parameters can determine a plane.

Since the roadway floor is rugged, it is not possible to construct a pose graph with a fixed plane as a node for optimization. In order to ensure that the constructed pose graph conforms to the actual situation, the roadway plane extraction is proposed based on the sub-map. According to the constructed local point cloud map, the tunnel plane parameters $\pi_{m}=[n_{a},n_{b},n_{c},d]$ are extracted. In order to ensure the efficiency of the roadway plane extraction, the current position is taken as the origin, and the point cloud is searched in the sub-map within the radius of the laser radar’s measured distance. According to the 3D LiDAR pose $p_{t}$ at this moment, the tunnel plane parameters $\pi_{m}$ are converted to the sensor local coordinate system by using Formulas (9) and (10):

(9) $${\left[n_{a}^{\prime },n_{b}^{\prime },n_{c}^{\prime }\right]}^{T}=R_{t}\cdot {\left[n_{a},n_{b},n_{c}\right]}^{T},$$

(10) $$d^{\prime }=d-t_{t}\cdot {\left[n_{a}^{\prime },n_{b}^{\prime },n_{c}^{\prime }\right]}^{T},$$

where $\pi_{m}^{\prime }=[n_{a}^{\prime },n_{b}^{\prime },n_{c}^{\prime },d^{\prime }]$ is the coordinate of the roadway plane in the sensor coordinate system, and $\left[R_{t},t_{t}\right]$ is the sensor pose at t.

The error between the pose node and the tunnel plane node [30] is calculated as Formula (11):

(11) $$e_{i,m}=q(\pi_{m}^{\prime })-q(\pi_{t})$$

where $q(\pi )=[\arctan(\frac{n_{a}}{n_{b}}),\arctan(\frac{n_{c}}{\left|n\right|}),d]$.

#### 3.4.3. Graph Optimization Construction

The graph optimization with sensor poses and spatial points is called Bundle Adjustment (BA), which can effectively solve large-scale positioning and mapping problems. However, as time goes by, the trajectory of mining equipment will become longer and longer, and the map scale will continue to grow. The BA method will reduce the computational efficiency. The pose graph optimization provides a new idea to solve this problem, and only regards them as the constraint of pose estimation, and no longer optimizes the pose estimation of feature points.

**Figure 6:**
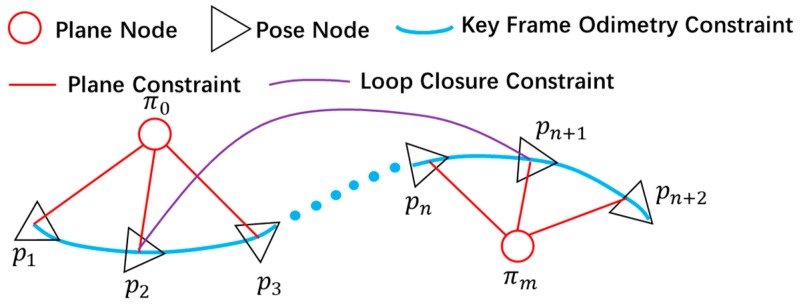
Schematic diagram of the pose structure SLAM, $\left\{p_{1},p_{2},\ldots p_{n+2}\right\}$ is the key frame pose node, $\left\{\pi_{0},\pi_{1},\ldots \pi_{n}\right\}$ is the extracted roadway ground plane coefficient node.

Based on the pose graph optimization theory, based on the calculated key point pose, plane constraint and loop detection constraints, the construction of the graph SLAM of the underground mining environment is constructed, as shown in Figure 6.

The pose graph optimization problem can be effectively solved by standard optimization methods, such as the Gauss-Newton or Levenberg Marquardt (LM) [31] algorithm. It has already integrated in the optimization library General Graph Optimization(G2O). To do this, it is only necessary to construct the corresponding pose nodes, loop nodes and plane nodes and their corresponding edges. According to the above laser consecutive key frames odometer, loop detection and roadways plane detection, corresponding nodes and edges can be constructed, and the G2O optimization library [32] can be used to solve the pose graph optimization problem.

### 3.5. Point Cloud Map Construction

The mapping frequency is lower than the laser odometer frequency. According to the above laser consecutive key frame odometer, when a frame point cloud is detected as a key frame, the underground environment map needs to be updated. The main method is to convert the real-time point cloud of the key frame into the world coordinate system through coordinate transformation, and compress the point cloud through the octree structure [33].

**Figure 7:**
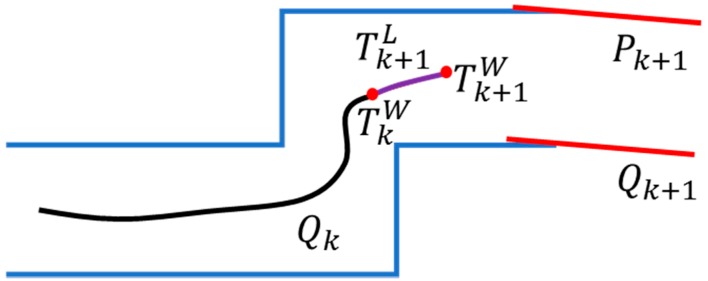
Point cloud map construction diagram.

When the k+1 frame is selected as the key frame, the relative pose $T_{k+1}^{L}$ between the key frames is obtained by using the GICP for continuous key frame optimization. According to the pose $T_{k}^{W}$ of the k key frame in the world coordinates. The pose $T_{k+1}^{W}=T_{k}^{W}T_{k+1}^{L}$ of the k+1 key frame in the world coordinates can be obtained by the coordinate transformation. Finally, the point cloud coordinates of the k+1 key frame are converted to the coordinate $Q_{k+1}$ in the world coordinate system by $T_{k+1}^{W}$, and the point cloud map is updated by the octree structure. As shown in Figure 7.

**Figure 8:**
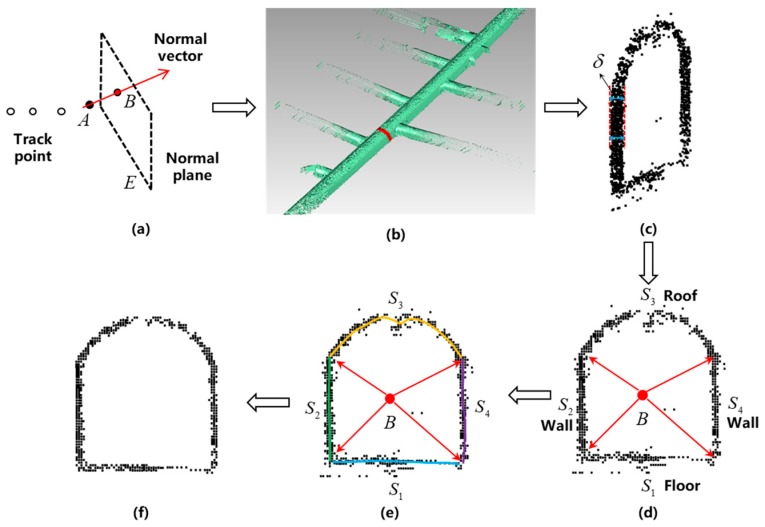
Roadway point cloud noise removal flow diagram.

In the process of constructing the point cloud map, when the key frame is added to the sub-map, the point cloud noise at the pose is removed according to the characteristics of the underground roadway to ensure that the constructed map satisfies the actual situation as much as possible. At the same time, it is also possible to remove moving objects such as pedestrians, so that the registration and positioning results are more reliable. As shown in Figure 8. The specific steps for removing noise from the roadway point cloud are as follows:

Step 1: The line connecting the current track point with the previous track point is taken as the normal vector, and the calculation method is as follows: two track points $A(x_{1},y_{1},z_{1})$, $B(x_{2},y_{2},z_{2})$ are known. Vector $\vec{a}=\vec{OA}=(x_{1},y_{1},z_{1})$, $\vec{b}=\vec{OB}=(x_{2},y_{2},z_{2})$. The normal vector is $\vec{AB}=\vec{OB}-\vec{OA}=(x_{2}-x_{1},y_{2}-y_{1},z_{2}-z_{1})$. Moreover, the normal plane equation at the track point is calculated according to the normal vector by using Formula (12), as shown in Figure 8a.

(12) $$(x_{2}-x_{1})(x-x_{2})+(y_{2}-y_{1})(y-y_{2})+(z_{2}-z_{1})(z-z_{2})=0.$$

However, considering the robustness and trajectory of the algorithm, when the following points occur in the trajectory point, noise removal processing is not performed here:

1. Calculate the curvature of the point, and exclude the calculation as a normal vector in the case of large fluctuations in curvature;
2. Calculating the distance between the track point and the previous point. When the distance is greater than a certain threshold, the point is excluded from the normal vector calculation;
3. Calculate the angle between the line connecting the track point and the previous point and the *X* axis, and compare the relationship between the angle and the heading angle of the point. When it is greater than a certain threshold, the point is excluded from the normal vector calculation.

Step 2: Based on the given section intercepting the thickness δ, the point cloud band with the bandwidth $\frac{\delta }{2}$ on both sides of the normal plane is obtained. That is, calculate the Euclidean distance from the point in the point cloud to the normal plane, which satisfies the Formula (13), as shown in Figure 8b,c.

(13) $$d_{i}\leq \frac{\delta }{2}.$$

Step 3: The point cloud is considered to be on the same plane, and the points that do not fall on the plane are projected, thereby obtaining a discrete point set of the normal plane. At the same time, in order to ensure the noise removal effect, according to the mine roadway design standard, the roadway structure is divided into a roof, a floor and two walls, and the point cloud of the roadway section is divided into four parts $S_{1},S_{2},S_{3},S_{4}$, as shown in Figure 8d,e.

Step 4: After obtaining the distribution of the roadway segment, the RANSAC method is first used to fit the curve, then the inner point is fitted by the least squares method, and finally the outer point of each part is removed to obtain the noise removal roadway section point cloud, as shown in Figure 8f.

In the process of constructing the point cloud map, the above four steps are repeated for each track point, and the roadway point cloud noise is automatically removed.

## 4. Experiment

### 4.1. Introduction to the Experimental Platform

**Figure 9:**
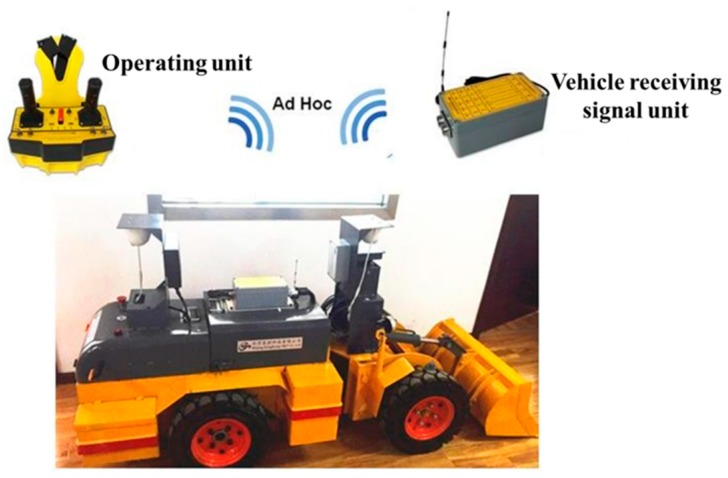
Underground mine trackless equipment experimental platform.

The unmanned operation experiment and development platform of the scraper is made according to the real vehicle size 1:5. Its function is basically the same as that of the real car. It consists of a bucket, a boom, a connecting rod, a rocker arm, a rotary cylinder, and a lifting cylinder. It realizes the sight-seeing remote control of the scraper. It can monitor the operating conditions of the scraper, including information such as pressure, stroke, speed, and angle of rotation to meet the experimental requirements of unmanned shovel loading. The platform vehicle supports the line control logic, which mainly includes the line control movement, the line control steering and the line control throttle. It provides an experimental platform for the independent operation of the scraper, and supports the line control walking and line control loading and unloading of the scraper through the software development platform, as shown in Figure 9.

**Figure 10:**
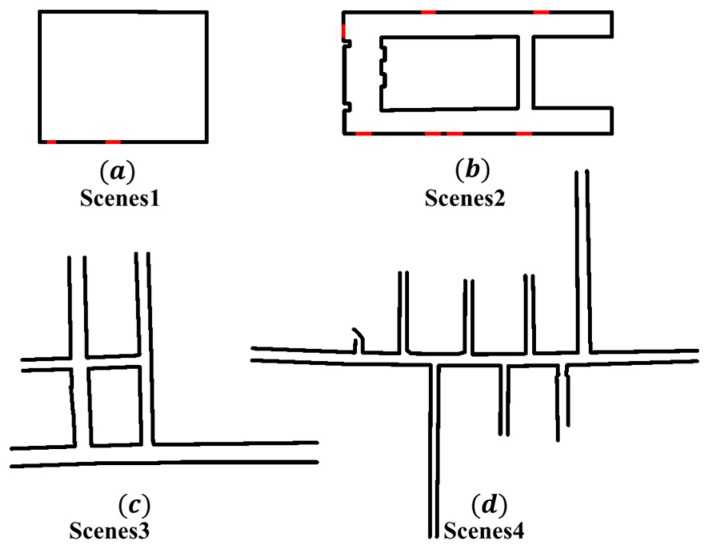
Schematic diagram of four scenes of structured corridors and mine roadways.

This experiment only uses Velodyne16 as a sensor. The experimental scene includes four scenes of structured corridor and mine roadway, as shown in Figure 10. Figure 10a is a structured office that has not been renovated. Figure 10b for the office corridor. Figure 10c shows the roadway with ring. Figure 10d shows the scene of the mine main roadway. Experiment and analyze the four localization and mapping methods of LOAM [19], Lightweight and Ground-Optimized Lidar Odometry and Mapping(LEGO-LOAM) [20], Berkeley Localization And Mapping(BLAM) [34] and GICP-SLAM for four scenarios. The goal of the LOAM is to build a real-time laser odometer using a two-axis LiDAR that moves in three dimensions. The distortion effect caused by the movement of the LiDAR is eliminated. Based on the core idea of separating localization and mapping, one is to perform high-frequency odometers but low-precision motion estimation (localization), and the other is to perform matching and register point cloud information at an order of magnitude lower frequency (mapping and correcting odometers). A high-precision and real-time laser odometer is obtained by combining the two parts; The author of LEGO-LOAM proposed a lightweight and ground-optimized LiDAR odometer and mapping method for estimating the six-degree-of-freedom attitude of a ground vehicle in real time. First, point cloud segmentation is applied to filter out noise and feature extraction to obtain unique planar and edge features. Then, using the two-step Levenberg-Marquardt optimization method, the planar and edge features are used to solve the different components of the six degrees of freedom transform in the continuous scan. A velodneVLP16 laser was used in the BLAM system. First, the input point cloud data is filtered. Then, the pose transformation of the two-frame point cloud data is calculated by the GICP algorithm, the nearest point in the map corresponding to the current frame is obtained according to the obtained initial pose transformation result, and GICP is performed again to obtain an accurate pose transformation. Finally, the point cloud data of the current frame is compared with the historical data to determine whether a loop occurs, and the pose and the point cloud map are published. The corresponding localization and mapping algorithm is implemented in the Ubuntu16.04 system of i7-8700CPU based on the robot operating system.

### 4.2. Results

**Figure 11:**
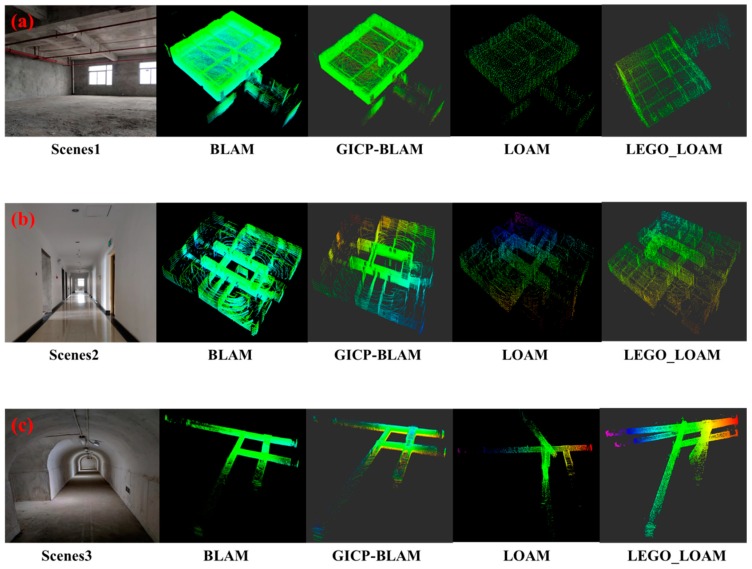

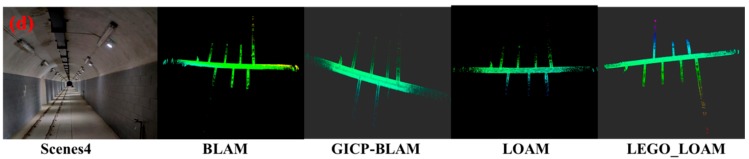
(**a**) Shows the indoor scene of an office that has not been renovated and the point cloud map constructed using the four SLAM methods; (**b**) the office corridor scene and the point cloud map constructed using the four SLAM methods; (**c**) shows the “ring” type underground roadway scene and the point cloud map constructed by using four SLAM methods; (**d**) is the underground main roadway scene and the point cloud map constructed by using four SLAM methods.

For the four experimental scenarios, the experiment was executed under the same conditions. The input of the algorithm only contains the 3D laser information. The sensor information, such as IMU, is not provided as the pose prediction. Furthermore, the effects of BLAM, GICP-SLAM, LOAM, and LEGO-LOAM are analyzed and compared. The specific mapping effect is shown in Figure 11a–d.

According to the point cloud map of Figure 11, the four SLAM mapping methods can construct a complete point cloud map, which is no big difference in macro for the indoor scene of the office building in Figure 11a and the office corridor scene of Figure 11b. For the “ring” type scenario of underground roadway, the point cloud map constructed based on the BLAM and the GICP-SLAM proposed in this paper is relatively complete. However, the “ring” type roadway map cannot be constructed based on LEGO-LOAM and LOAM. These methods have higher requirements on the initial pose. Because there is no IMU-like sensor for pose estimation, the cumulative error of the pose is gradually increased. At the same time, the characteristics of the underground tunnel scene are relatively small, and the feature extraction cannot be performed well. The construction of the point cloud map failed; for the underground main roadway scene, the system cumulative error did not show large fluctuations due to no turning, and the four SLAM methods can construct the completed point cloud map.

**Figure 12:**
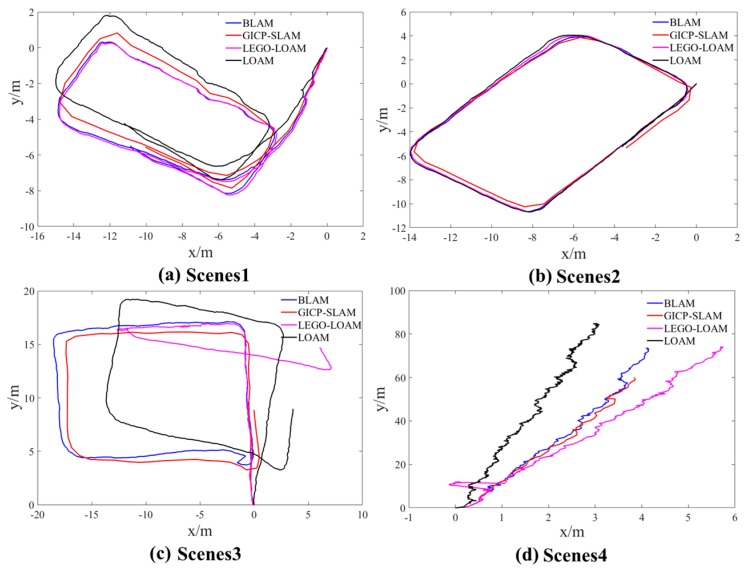
(**a**) Shows the trajectory of the four SLAM methods in the interior scene of the office building that has not been renovated; (**b**) shows the trajectory of the four SLAM methods in the office corridor scene; (**c**) shows the four SLAM methods in the running track of the “ring” type scene of the underground roadway; (**d**) shows the running track of the four SLAM methods in the underground main roadway scene.

In the experiment process, in addition to the construction of the point cloud map in the four scenarios, the pose of the experimental equipment in the four scenarios is also calculated. The trajectories of the four SLAM methods in the four scenarios are shown in Figure 12a–d.

In the indoor and underground roadway scenes, due to the experiment conditions, the actual trajectory of the equipment cannot be accurately measured. According to Figure 12, the relative positioning of the four SLAM methods in the four scenarios can be compared. For the indoor scenes of uneven ground office buildings, the positioning results of the four SLAM methods are similar, but the positioning deviation based on the LOAM method is larger than other methods; for the flat office corridor scene, the positioning trajectories of the four SLAM methods are almost coincident, and the overall positioning effect is better. According to the BLAM method and the GICP-SLAM method proposed in this paper, the overall positioning results are similar for the roadway scene with rough ground and inconspicuous features. However, based on the results, the LOAM and LEGO-LOAM methods have the same situation as the mapping, that is, positioning deviation occurs. The results are explained in the section on mapping results.

In the four scene experiments, the four SLAM methods of each scene have the same starting point and ending point. The starting position is set to the origin and there is no rotation. By analyzing the relative translation of the starting point and the ending point under the four SLAM methods of the four scenes. The relative deviations of the relative rotations are used to quantitatively analyze the positioning results of the four methods, as shown in Table 1. It can be seen from the table that the total relative translation and the total relative rotation of the four SLAM methods in the four scenarios are basically the same for the first scenario. The positioning results of the SLAM method are consistent, but for the underground roadway scene, the deviation of the translation and rotation based on LEGO-LOAM and LOAM is also large, which also shows that it is difficult to obtain accurate positioning results by relying only on the three-dimensional laser information. However, the GICP-SLAM method proposed in this paper can still achieve more accurate localization and mapping in the case of only three-dimensional laser information.

### 4.3. Discussion of Results

In order to further analyze the localization and mapping effect of the GICP-SLAM algorithm proposed in this paper. The effects of the four modules of the algorithm were analyzed, licluding: point cloud registration, loop constraint, plane constraint, and running time.

#### 4.3.1. Impact of Point Cloud Registration

**Figure 13:**
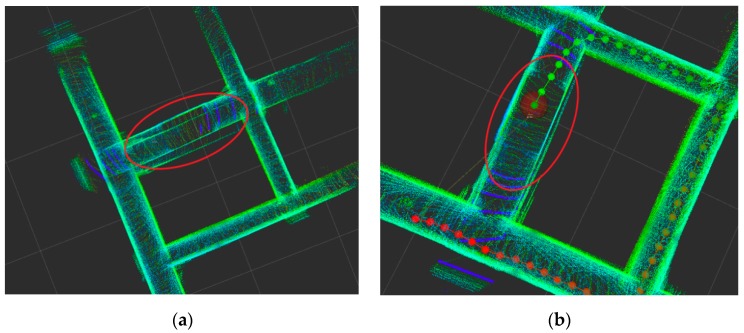
Comparison of point cloud registration based on (**a**) Normal Distributions Transform (NDT) and (**b**) GICP.

Through experimental analysis, different point cloud registration algorithms have a greater impact on their mapping, as shown in Figure 13. Figure 13a is a point cloud registration based on NDT. The point cloud registration accumulative error is larger from the red circle circled in the figure. There is a large deviation after the corner, which requires loop detection for further correction. Figure 13b shows the GICP based point cloud registration map. It can be seen that the GICP registration error is smaller in the same position. The point cloud map constructed by it has less deviation, and the point cloud is more consistent with the local map of the point cloud, as shown in the red circle in Figure 13b. The quasi-method has a great influence on the construction effect. It can be seen that the point cloud registration method has a great influence on the mapping effect. The registration accuracy based on GICP is better, and it is better adapted to the underground mine production environment.

#### 4.3.2. Impact of Loop Constraints

**Figure 14:**
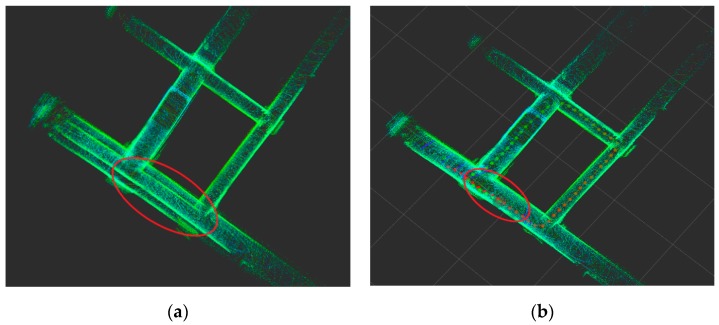
Effect of loop detection constraints on localization and mapping in pose graph. (**a**) Without loop detection, (**b**) with loop detection.

Through experimental analysis, the loop constraint of the pose graph optimization has a great influence on the map construction, as shown in Figure 14. Figure 14a does not add loop constraint in graph optimization, which leads to a large deviation of the constructed point cloud map. Figure 14b adds loop constraint in graph optimization. The cumulative error of the odometer is corrected, and finally, a globally consistent point cloud map is constructed.

#### 4.3.3. Influence of Plane Constraints

**Figure 15:**
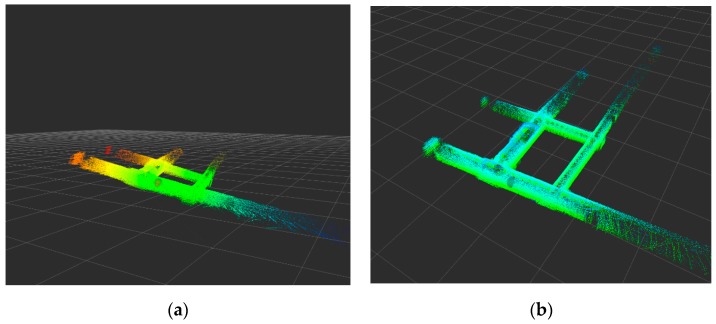
Effect of plane detection constraints on localization and mapping in pose graph. (**a**) Without loop detection, (**b**) with loop detection.

Through experimental analysis, the plane constraint in the pose map also has a great influence on the mapping result. As shown in Figure 15. Figure 15a does not add the plane detection constraint in the pose graph. The constructed point cloud map cannot be corrected, so that it is inclined at a certain angle. Figure 15b adds a plane detection constraint in the pose graph. The cumulative error in the process of positioning and mapping is corrected, and finally a globally consistent point cloud map is constructed.

#### 4.3.4. Run Time Analysis

In order to explore the real-time performance of the GICP-SLAM algorithm, the experiment was carried out in the underground ring roadway to analyze the running time of the main modules such as plane detection, laser odometer, loop detection, and graph optimization. See Table 2 for details. According to the table, for the laser point cloud plane extraction, the average time per frame is only about 10 ms, and there is no big deviation in the whole process. For the GICP laser point cloud odometer, it takes about 51 ms per frame to perform point cloud registration, and the longest needs 111 ms iterative matching to converge; Since loop detection requires loop judgment, and it is also necessary to perform GICP-based registration between loop frames for pose optimization, so the average loop time is about 114 ms; The averaging time of the pose graph optimization is about 15 ms, and the convergence condition can be quickly satisfied.

## 5. Conclusions

A 3D GICP-SLAM method based on underground mining environment was proposed. The registration between point cloud consecutive frames, consecutive key frames and loop frames was carried out based on GICP. Innovative extraction of roadway planes and loops that was detected and optimized based on rules and GICP as graph optimization constraints, reducing the cumulative error caused by laser odometers alone. For the constructed sub-map, the point cloud noise is automatically removed based on the characteristics of the roadway.

The proposed method was evaluated in four scenarios (such as the underground mine laboratory) and compared with the existing 3D laser SLAM method (such as LOAM). The results showed that the algorithm could realize low drift localization and point cloud map construction. This method provides technical support for real-time localization and navigation of underground mining environment.

The effects of the main functional modules of GICP-SLAM algorithm on the localization and mapping of underground environment were analyzed from four aspects: point cloud odometer registration, loop detection, plane constraints, and system module running time. Explain the rationality of the algorithm.

In the subsequent work, the underground mine scene will be further combined to carry out research on multi-sensor fusion localization and mapping based on IMU, so that the positioning and mapping accuracy will be further improved. At the same time, the storage method of point cloud map and the type of data stored are studied, which is the basis for real-time localization of underground mining environments based on maps.

## Figures and Tables

**Table 1:** Relative deviation results of translation and rotation of the same starting point and end point under four SLAM methods in four scenarios.

Scenes	Method	Trans.^1^ *X*	Trans. *Y*	Trans. *Z*	Total Trans. (m)	Roll	Pitch	Yaw	Total Rotat. ^2^ (rad)
1	Blam	−10.88	−5.50	0.19	12.19	−0.02	0.05	−1.42	1.42
	Gicp_Slam	−10.04	−5.56	1.46	11.57	−0.01	0.03	−1.42	1.42
	Lego_Loam	−10.78	−5.64	0.14	12.17	−0.16	−1.41	−0.17	1.43
	Loam	−11.20	−4.23	0.21	11.97	−0.49	−1.51	−0.50	1.67
2	Blam	−3.64	−5.25	−0.12	6.38	0.00	0.00	0.02	0.02
	Gicp_Slam	−3.44	−5.35	1.45	6.52	0.00	0.02	−0.09	0.09
	Lego_Loam	−3.61	−5.22	−0.01	6.35	−0.04	0.02	−0.04	0.06
	Loam	−3.61	−5.23	−0.09	6.35	0.00	0.02	0.00	0.02
3	Blam	−0.57	9.57	0.40	9.59	0.01	0.03	0.46	0.46
	Gicp_Slam	−0.01	8.88	1.22	8.96	−0.03	−0.02	0.37	0.37
	Lego_Loam	6.05	14.71	−3.51	16.28	−0.61	0.04	1.14	1.29
	Loam	3.57	8.94	−3.13	10.13	0.31	0.24	−0.04	0.39
4	Blam	4.09	73.50	3.77	73.71	0.05	−0.02	0.19	0.20
	Gicp_Slam	3.84	60.04	1.28	60.18	0.01	−0.03	0.07	0.07
	Lego_Loam	5.70	73.96	2.96	74.24	−0.06	0.15	0.03	0.17
	Loam	2.99	84.75	3.85	84.89	−0.01	0.19	0.05	0.20

^1^ Trans.: Translation; ^2^ Rotat.: Rotation.

**Table 2:** GICP-SLAM module runtime.

Model	Max (ms)	Min (ms)	Mean (ms)
Planar Detection	10.13	10.04	10.09
LiDAR Odometry	111.00	10.09	51.22
Loop Detection	252.13	80.59	114.67
Graph Optimization	50.41	10.05	14.39
